# iCpG-Pos: an accurate computational approach for identification of CpG sites using positional features on single-cell whole genome sequence data

**DOI:** 10.1093/bioinformatics/btad474

**Published:** 2023-08-09

**Authors:** Sehi Park, Mobeen Ur Rehman, Farman Ullah, Hilal Tayara, Kil To Chong

**Affiliations:** Department of Electronics and Information Engineering, Jeonbuk National University, Jeonju 54896, South Korea; Department of Electronics and Information Engineering, Jeonbuk National University, Jeonju 54896, South Korea; College of Information Technology in the United Arab Emirates University (UAEU), Abu Dhabi 15551, UAE; School of International Engineering and Science, Jeonbuk National University, Jeonju 54896, South Korea; Department of Electronics and Information Engineering, Jeonbuk National University, Jeonju 54896, South Korea; Advances Electronics and Information Research Center, Jeonbuk National University, Jeonju 54896, South Korea

## Abstract

**Motivation:**

The investigation of DNA methylation can shed light on the processes underlying human well-being and help determine overall human health. However, insufficient coverage makes it challenging to implement single-stranded DNA methylation sequencing technologies, highlighting the need for an efficient prediction model. Models are required to create an understanding of the underlying biological systems and to project single-cell (methylated) data accurately.

**Results:**

In this study, we developed positional features for predicting CpG sites. Positional characteristics of the sequence are derived using data from CpG regions and the separation between nearby CpG sites. Multiple optimized classifiers and different ensemble learning approaches are evaluated. The OPTUNA framework is used to optimize the algorithms. The CatBoost algorithm followed by the stacking algorithm outperformed existing DNA methylation identifiers.

**Availability and implementation:**

The data and methodologies used in this study are openly accessible to the research community. Researchers can access the positional features and algorithms used for predicting CpG site methylation patterns. To achieve superior performance, we employed the CatBoost algorithm followed by the stacking algorithm, which outperformed existing DNA methylation identifiers. The proposed iCpG-Pos approach utilizes only positional features, resulting in a substantial reduction in computational complexity compared to other known approaches for detecting CpG site methylation patterns. In conclusion, our study introduces a novel approach, iCpG-Pos, for predicting CpG site methylation patterns. By focusing on positional features, our model offers both accuracy and efficiency, making it a promising tool for advancing DNA methylation research and its applications in human health and well-being.

## 1 Introduction

DNA methylation is a crucial epigenetic marker and performs an important role in a wide range of biological processes, such as chromosomal instability, cell differentiation, X-chromosome inactivation, cancer progression, and gene regulation (Robertson 2005, Suzuki and Bird 2008, Laird 2010, Jones 2012). DNA methylation is related to the functional state of regulatory regions and affects DNA replication and gene transcription. These features are closely connected to various human diseases such as immune diseases, malignant tumors, and Alzheimer’s (Gao *et al.* 2017, Wan *et al.* 2015, Stieglitz *et al.* 2017, Yan *et al.* 2017). Recent research has shown that methylation levels are closely linked to age and life expectancy (Smallwood *et al.* 2014). In particular, previous research has indicated that DNA methylation levels vary with age (Horvath 2013). Therefore, it is crucial to study DNA methylation.

Until now, well-established approaches have been developed to quantify average levels of DNA methylation in cell populations. Recent advances in technologies have made it possible to profile DNA methylation at single-cell resolution via reduced representation protocols (scRRBS-seq) (Smallwood *et al.* 2014) or whole genome bisulphite sequencing (scBS-seq) (Clark *et al.* 2017). These methods have disclosed the dynamics of epigenetic patterns in heterogeneous cell populations and enabled explaining epigenetic networks with unprecedented resolution and scale (Hou *et al.* 2016).

Since genetic material per cell is limited, analyzing single-cell methylation is inherently limited by moderate CpG coverage. Smallwood *et al.* (2014) found that the scBS-seq method covers 20%–40% of CpG sites, while the scRBS-seq method only covers 1%–10% of CpG sites (Farlik *et al.* 2015, Guo *et al.* 2013, Hou *et al.* 2016). A decrease in CpG coverage could result in the loss of critical information. Therefore, the first critical step is to identify unknown methylation states to enable whole-genome analyses.

Various approaches have been proposed to detect the average DNA methylation profiles in cell populations (Zhang *et al.* 2015, Stevens *et al.* 2013, Ernst and Kellis 2015, Liu *et al.* 2015, Whitaker *et al.* 2015). However, these methods fail to explain cell-specific variability, and they require pre-defined features and genomic annotation, which are often limited to a narrow range of cell conditions and types. Therefore, it is crucial to develop more efficient computational approaches to predict DNA methylation and make methylation identifications more reliable (Zhang *et al.* 2015).

Identifying methylation states in tissue samples is a well-known challenge in bioinformatics, and one approach is to leverage dependencies between CpG sites. For instance, some studies used variational autoencoders to reduce the dimensionality of methylation data (Levy *et al.* 2020). Other methods employ machine learning techniques such as random forests (RFs) (Zhang *et al.* 2015), autoencoders (Qiu *et al.* 2018), gradient boosting (Zou *et al.* 2018), linear regression (Di Lena *et al.* 2019), or mixture models (Yu *et al.* 2020) to impute individual CpG sites in tissue samples. Some of these approaches also consider intrasample dependencies between adjacent CpG sites and leverage information from multiple tissue samples to identify CpG sites (Zou *et al.* 2018, Yu *et al.* 2020).

Most recent studies on DNA methylation imputation in single cells have leveraged both intercellular and intracellular correlations between methylation states. One such method is Melissa, which first identifies a genomic region of interest (e.g. a specific promoter region) and then performs generalized linear model regression on that region to identify CpG sites (Kapourani and Sanguinetti 2019). This model effectively clusters cells by leveraging information from other cells through a shared prior distribution determined by the Bayesian mixture model.

The nature of methylation sites must be vectorized so that computational models can identify them. A lot of previous studies (Zhou *et al.* 2012, Bhasin *et al.* 2005, Pavlovic *et al.* 2017) have shown that the sequence of adjacent nucleotides of a methylation site is specific and that the methylation states are closely associated with the sequence composition. Bhasin *et al.* (2005) proposed the Methylator method which used conventional binary sparse encoding to convert sequences directly into feature vectors. Das *et al.* (2006) developed a method that extracts sequences with a window size of 800 bp, counts the methylation propensity, and uses principal component analysis with recursive feature removal for feature selection. Recently, Pan *et al.* (2018) used an k-gram, multivariate reciprocal information (Ding *et al.* 2016), Discrete Wavelet Transform (Shensa *et al.* 1992), and Pseudo Amino Acid Composition (Chou 2001) to extract DNA sequence features for training sparse Bayesian learning model. All of these techniques have established that the model performance is directly dependent on the selection of the classifier and the feature extractor.

Zhang’s method proposes a RF based model to handle differences in local CpG profiles between different cells (Zhang *et al.* 2015). Zhang’s technique trained the RF classifier using four factors: chromosomal location features, DNA sequence features, cis-regulatory elements, and the levels and lengths between adjacent CpG sites.

Further, another model LightCpG was proposed to identify CpG sites (Jiang *et al.* 2019). The LightCpG model uses novel positional features, structural features and n-gram features to train the model. We are highly inspired by the positional features used in the LightCpG architecture, however, we believe the potential of these positional features is still unexplored. Therefore, in this research, we propose an ensemble learning-based method using positional features to predict the methylation sites. Achieved results have demonstrated that the proposed model outperformed existing techniques and a significant improvement is observed.

## 2 Materials and methods

**Figure 1.**
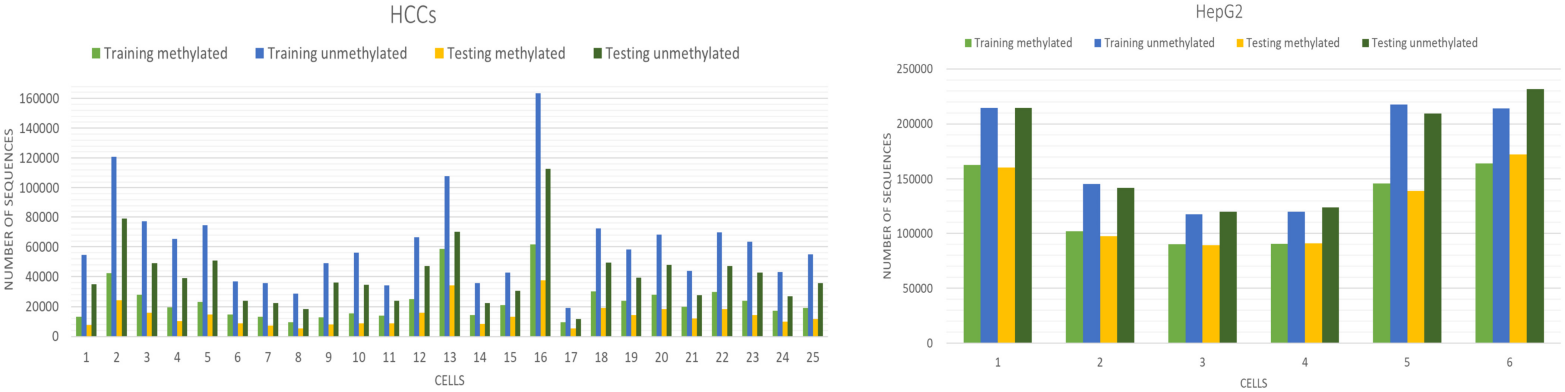
Cell-wise methylated and non-methylated, training, and testing data size visualization of HCCs and HepG2 datasets.

As can be seen in Fig. 1, there are three major milestones in the creation and evaluation of iCpG-Pos. The first step is to collect the dataset which is obtained from the literature (Angermueller *et al.* 2017). The second step is model construction which is the biggest milestone. The model construction consists of feature extraction, classifier selection, and ensemble learning strategy selection. Selection of the optimized end-to-end model is a critical step towards the development. The last milestone is to evaluate the different models and propose the best model.

### 2.1 Dataset

Similar to LightCpG, we have used two benchmark datasets. The first dataset (GSE65364) consists of 25 human hepatocellular carcinoma cells (HCCs). Though there are 26 HCCs but Ca26 is eliminated since the pattern of transcriptional activity is substantially anomalous, according to the work by Hou *et al.* (2016). The second dataset contains six human hepatoblastoma-derived (HepG2) cells attained from (GSE65364). Both datasets were profiled using single-cell reduced-representation-bisulphite-sequencing (scRRBS-seq). Using the liftOver tool (http://www.genome.ucsc.edu/cgi-bin/hgLiftOver), each location of a CpG site is aligned to hg19 (Raney *et al.* 2014). In our study, we looked at these sites as research items that had at least four reads on them.

Figure 1 visualizes the amount of data in each cell of HCCs and HepG2 datasets used for training and testing. We applied the same validation strategy across all datasets, drawing inspiration from the LightCpG. Table 1 shows the dataset distribution for training, testing, and validation.

**Table 1.** Dataset distribution for training, testing, and validation sets.

Dataset	Set	Chromosomes
**HCCs/HepG2**	Train	1,3,5,7,9,11
	Test	2,4,6,8,10,12
	Validation	13,14,15,16,17,18,19

### 2.2 Feature extraction

We have used three feature encoding/extraction techniques to decide the best among them. These techniques are one-hot encoding, n-gram and positional features. Herein, these features are individually used towards the development of iCpG-Pos. These feature encoding techniques are discussed below.

#### 2.2.1 One-hot encoding

The one-hot encoding strategy is the most basic and widely used encoding technique utilized in computational biology (Rehman *et al.* 2022b,c). In this encoding system, each nucleotide is translated to a numerical value, which is then allocated a specific binary vector that contains all “0” values except the integer’s index, which is maintained as “1.” The one-hot encoding for four nucleotides in a DNA sequence looks like this:


$$\begin{array}{c} A\Rightarrow [1,0,0,0]\\ T\Rightarrow [0,1,0,0]\\ G\Rightarrow [0,0,1,0]\\ C\Rightarrow [0,0,0,1] \end{array}$$

#### 2.2.2 n-gram features

To better know the importance of the positional features, we included the sequence feature. To get the sequence features we have used n-gram features. N-gram feature extraction entails extracting continuous sequences of n elements from a given sequence, such as words or letters (Ganapathiraju *et al.* 2002). These sequences, known as n-grams, can give useful information about the sequence data’s local context and sequential relationships. The sequence is tokenized into distinct parts, in order to extract n-gram characteristics. The set of n-grams is then formed by generating all feasible n-length sequences. The frequency of each occurrence of an n-gram is frequently estimated to illustrate its value or relevance within the sequence.

Every n-gram feature is represented by a pair of values $\left(o_{i},t_{i}\right)$, where $o_{i}$ is a feature that may be retained as combination of “n” number of nucleotides while $t_{i}$ is the number of occurrences of $o_{i}$ being repeated in the given sequence. $t_{i}$ is calculated by the following equation, where $N(o_{i})$ is the number of times $o_{i}$ is repeated in the sequence while M represents the length of the input sequence. In this research work, we have taken n to be 1, 2, and 3 to represent a single sequence. The final attained feature vector is of length 84.


(1) $$t_{i}=\frac{N(o_{i})}{M-(n-1)},$$

#### 2.2.3 Positional features

Applying knowledge regarding the CpG regions and the separation between nearby CpG sites, the authors of LightCpG proposed a method to derive positional characteristics of CpG sites (Jiang *et al.* 2019). To extract positional features, the states of both the target CpG site and its adjacent CpG sites, as well as the distance between them, are taken into consideration. Meaningful information about the states along with finding the distance among adjacent sites is required to extract positional features.

A new method termed as skip K method is utilized to determine the correlation between two adjacent CpG sites. The skip-K method comprises of two subparts “skip-$K_{1}$” and “skip-$K_{2}$,” they refer to different steps in this analysis. “skip-$K_{1}$” is the preliminary step that checks for the existence of similarity between adjacent sites. Once similarity has been established between adjacent CpG sites, the “skip-$K_{2}$” analyzes the extent of similarity between CpG sites within a certain window size.

The skip-$K_{1}$ method is a technique that extracts features from all CpG sites at specific distances from the target CpG site. It selects one CpG site at the upstream end which is located at the $K_{1}^{th}$ distance from the reference point (i.e. the target CpG site) and another CpG site at the downstream end which is also at the $K_{1}^{th}$ distance from the reference point. Once these CpG sites are extracted, the states are determined using the skip-$K_{1}$ method. The method then calculates the distance between the extracted states and the target CpG site. As the distance between the CpG sites increases, the correlation between adjacent sites becomes weaker.

The skip-$K_{2}$ method extracts features from CpG sites located at a specific distance from the target CpG site. This involves identifying the CpG sites closest to the target CpG site at a distance of $K_{2}$ from both the upstream and downstream ends. The method then determines the states of these extracted CpG sites and calculates the distance between the states and the target CpG site. Using this information, the method predicts the state of the target CpG site. It is important to note that as the window size increases, the prediction accuracy of the skip-$K_{2}$ method tends to decrease. Therefore, in order to ensure optimal prediction accuracy, the size of both $K_{1}$ and $K_{2}$ is set to 1.

In addition to that, the skip-$K_{1}$ and skip-$K_{2}$ methods, one CpG site state is also individually extracted from both the upstream and downstream ends near the target site. The states of these two CpG sites are then determined and the distance between these extracted states and the target site is calculated. This information is used to predict the state of the target site.

To handle the challenge of extracting features from CpG sites with similar methylation states that exist in different cells, we adopt the following approach. Let us assume there are *w* cells in the dataset. For each chromosome in the $t^{th}$ cell, we define a set $Y_{t}$ consisting of *n* CpG sites. This set can be represented as follows:


(2) $$Y_{t}=\left\{\left(p_{t}^{1},s_{t}^{1}\right),\left(p_{t}^{2},s_{t}^{2}\right),\ldots ,\left(p_{t}^{c},s_{t}^{c}\right),\ldots \left(p_{t}^{n},s_{t}^{n}\right)\right\}.$$

Here $p_{t}^{c}$ and $s_{t}^{c}$ refer to the position and methylation state of the $c^{th}$ CpG site in the $t^{th}$ cell, respectively.

With this approach, we can extract the features of CpG sites that are similar to one another across different cells. This enables us to capture the patterns of methylation states across multiple cells, which can improve the accuracy of our predictions.

To maximize the number of features extracted from sites that are similar but exist in different cells, we use $Y_{t}$ to devise methods for feature extraction. If a CpG site exists in the $r^{th}$ cell and satisfies the condition $p_{t}^{c}=p_{r}^{l}$, then we denote $F_{t,r}^{c}$ as the distance between the nearest CpG sites with methylation states on both ends of the CpG site in question, where the nearest CpG site is the one in the $l^{th}$ position in the $j^{th}$ cell.


(3) $$F_{t,r}^{c}=\left\{P_{t,r}^{l-1},S_{t,r}^{l-1},P_{t,r}^{l+1},S_{t,r}^{l+1}\right\}.$$

(4) $$\mathrm{Where},P_{t,r}^{l-1}=p_{r}^{l-1}-p_{t}^{c},P_{t,r}^{r+1}=p_{r}^{l+1}-p_{t}^{c},S_{t,r}^{l-1}=s_{t}^{l-1},S_{t,r}^{l+1}=s_{r}^{l+1}.$$

If a single CpG site exists in the *r*-th cell and has an unknown methylation status, then we select the two adjacent sites which satisfy the condition $p_{r}^{l}<p_{t}^{c}<p_{r}^{l+1}$. In this case, $F_{t,r}^{c}$ is denoted as follows:


(5) $$F_{t,r}^{c}=\left\{P_{t,r}^{l},S_{t,r}^{l},P_{t,r}^{l+1},S_{t,r}^{l+1}\right\}.$$

(6) $$\mathrm{Where},P_{t,r}^{l}=p_{r}^{l}-p_{t}^{c},P_{t,r}^{l+1}=p_{r}^{l+1}-p_{t}^{c},S_{t,r}^{l}=s_{r}^{l},S_{t,r}^{l+1}=s_{r}^{l+1}.$$

The features which are extracted for the CpG sites are similar in nature but they exist in different cells, these features are also represented and the following equation is used to extract feature vector,


(7) $$\begin{array}{c} D_{t,r}^{c}=\left\{P_{r,r}^{\left(l-2)(l-1\right)},S_{r,r}^{l-2},P_{r,r}^{\left(l-1)(l\right)},S_{r,r}^{l}\right\}\cup \\ \left\{P_{r,r}^{\left(l)(l+1\right)},\ldots S_{r,r}^{l},P_{r,r}^{\left(l+1)(l+2\right)},S_{r,r}^{l+2}\right\} \end{array}.$$

Here, $D_{t,r}^{c}$ represents the feature vector for the CpG site *c* in cell *t*, where *c* is similar to a CpG site in cell *r* with position $p_{r}^{l}$. If $p_{t}^{c}=p_{r}^{l}$ and t≠r, then the feature vector is given by the tuple $\left(P_{t,r}^{l},P_{t,r}^{l+1},S_{t,r}^{l}\right)$, which contains the distance to the nearest CpG sites on either side of site *c* in cell *r*, as well as the methylation state of the site *l* in cell *r*. If the condition is not satisfied, meaning the sites are not similar, then the feature is set to “unknown”.

The aim is to extract features of CpG sites that are similar in nature but exist in different cells. However, some of these CpG sites have unknown methylation states. To solve this, Q and H features are used for feature extraction. F represents two factors, namely the distance and methylation states of the two nearest CpG sites to a specific CpG site that satisfies the condition $p_{t}^{c}$=$p_{r}^{l}$ and is present in the *r*th cell. Following is the equation used in this case,


(8) $$\begin{array}{c} D_{t,r}^{c}=\left\{P_{r,r}^{\left(l-1)(l\right)},S_{r,r}^{l-1},P_{r,r}^{\left(l)(l+1\right)},S_{r,r}^{l+1}\right\}\cup \\ \left\{P_{r,r}^{\left(l)(l+1\right)},S_{r,r}^{l},P_{r,r}^{\left(l+1)(l+2\right)},S_{r,r}^{l+2}\right\} \end{array}.$$

**Figure 2.**
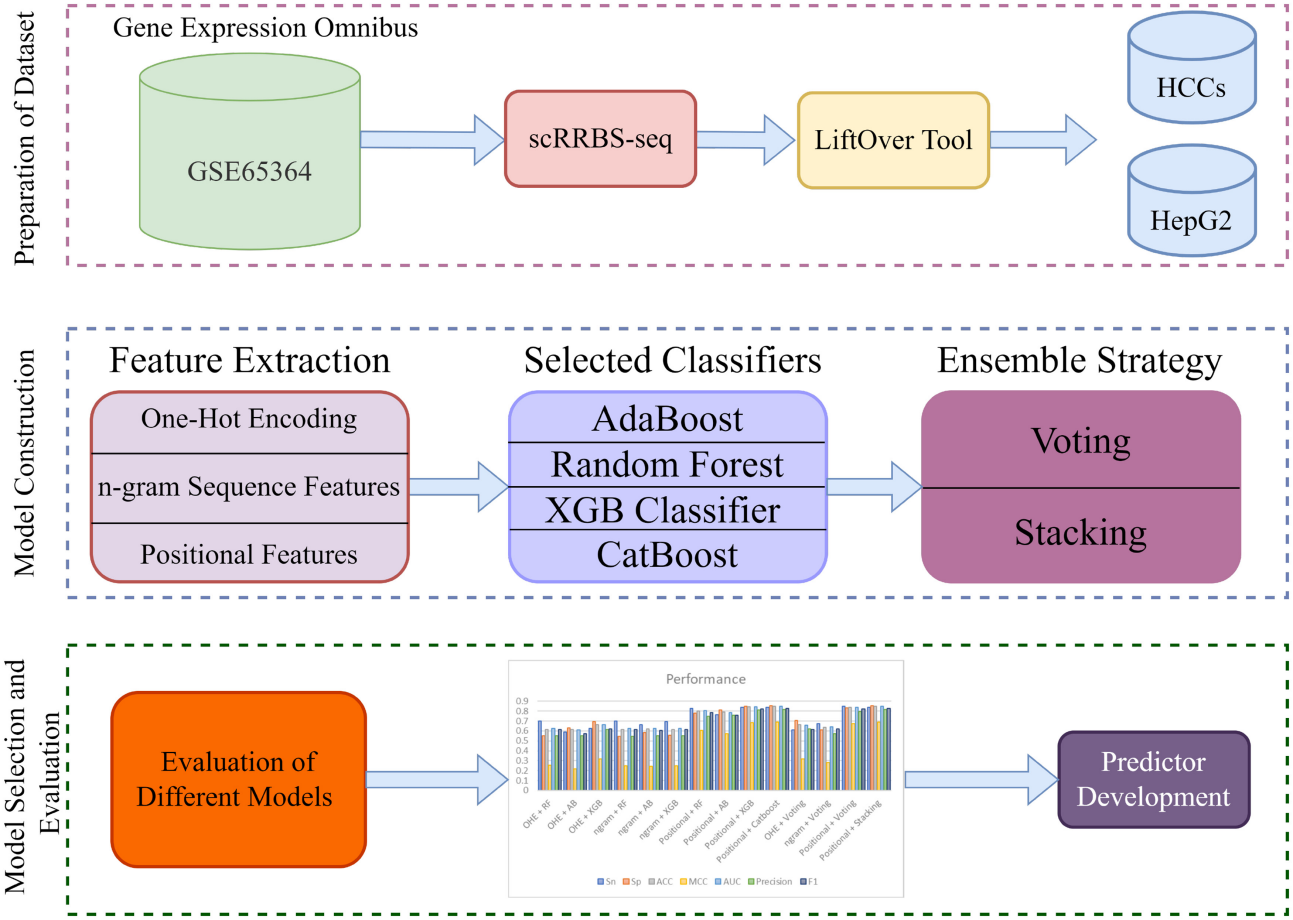
iCpG-Pos development flowchart in schematic form. It comprises of dataset creation, feature extraction, baseline model building, and stacking-predictor development.

*D* features are obtained when a single CpG site in the *r*th cell satisfies the condition $p_{t}^{c}$ =$p_{r}^{l}$ and *t* is not equal to *r*. In this case, *D* represents two factors, distance, and methylation state, of the CpG sites on both sides of the target CpG site. These CpG sites are (l−1) and (l + 1) sites. Figure 2 illustrates these details.

**Figure 3.**
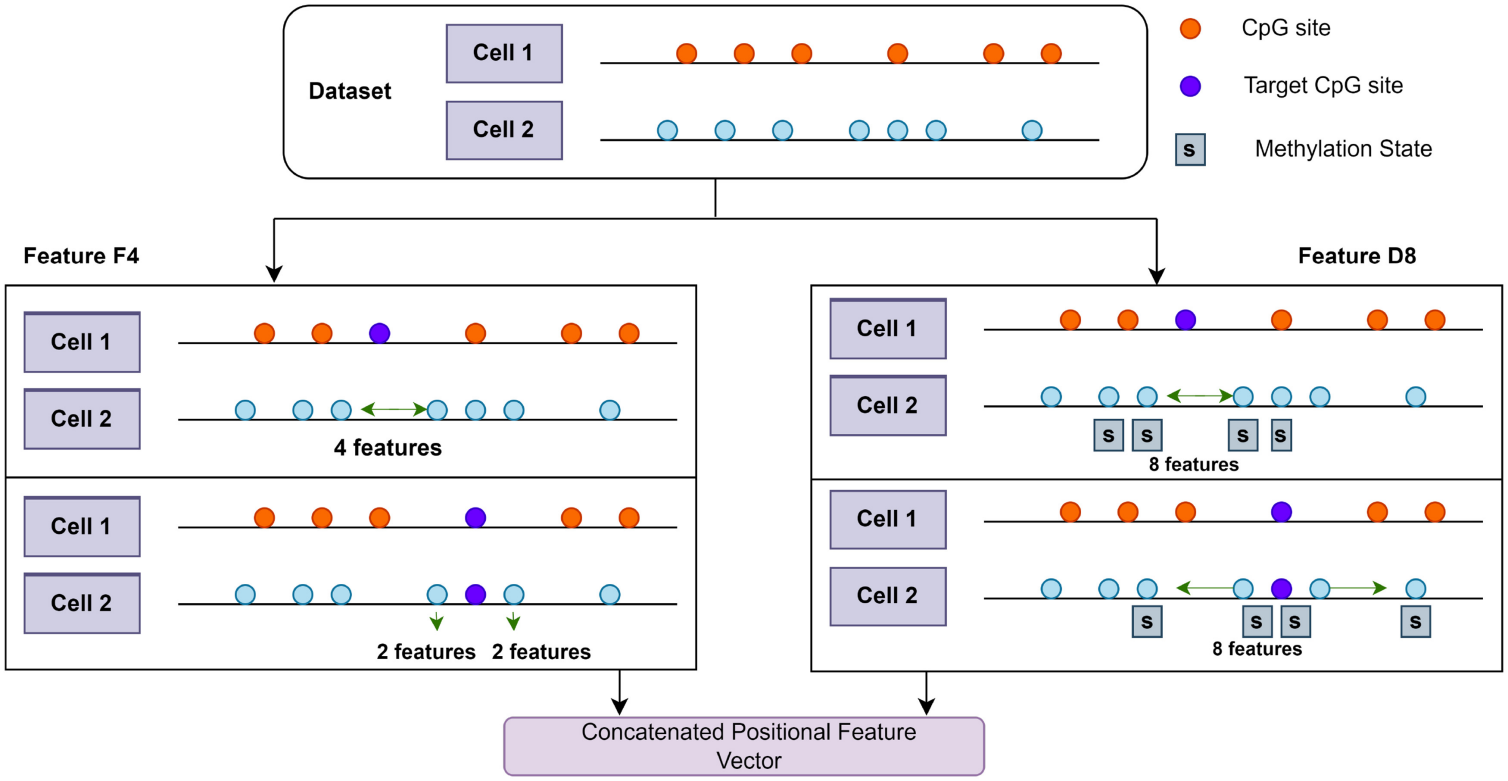
Visual illustration for extraction of positional features.

The features obtained are a combination of the distance and methylation states of the CpG sites. *F* and *D* features are represented by $F_{t,r}^{c}$ and $D_{t,r}^{c}$, respectively. Figure 3 shows the pictorial description of these details. Where $F_{t,r}^{c}$ represents the F4 features and $D_{t,r}^{c}$ represents the D8 features.

### 2.3 iCpG-Pos development

At the initial stage, we used weka software to select the baseline classifiers. Several classifiers were run on different features to get the list of best classifiers among all. The four selected classifiers are commonly used in Machine Learning techniques (AdaBoost, Random Forest, XGB, and CatBoost classifier). At first, each classifier is optimized on every feature vector individually. The optimization is performed using the OPTUNA algorithm. OPTUNA utilizes trial histories to identify which hyper-parameter variables to test subsequently (Akiba *et al.* 2019). It predicts a potential region and attempts parameters in that region while using the given range. According to the current finding, Optuna anticipates an even more competitive region. This technique repeatedly utilizes historical data from previous experiments. It implements a Bayesian optimization approach known as Tree-structured Parzen Estimator. The optimum hyper-parameter values for which the optimization algorithm Fit returns the best score. As a result, the fitness metric is calculated as follows: where ${\text{MCC}}_{i}$ represents the achieved Matthews correlation coefficient (MCC) on $j^{th}$ fold with the selected parameters. $N_{\mathrm{FCV}}$ represents the number of fold cross-validation which in our case is 5. The higher the value of Fit shows better the performance by the selected parameters.


(9) $$\text{Fit}=\frac{1}{N_{\mathrm{FCV}}}\sum_{j=1}^{N_{\mathrm{FCV}}}{\text{MCC}}_{j},$$

Intending to enhance CpG site prediction, we used a stacked and voting ensemble learning architecture to create the iCpG-Pos. Out of both the ensemble learning frameworks, the stacking algorithm exhibited to be better. Stacking, unlike the voting ensemble procedure, successfully examines and learns how to combine diverse baseline models to generate a more precise model. In the stacking algorithm, an extra-tree classifier (ETC) is used as a meta-predictor. To incorporate the distinct strengths of several baseline models, the ETC predictor is developed and optimized using the new feature vector. But it is being observed that the stacking algorithm showed almost similar results to the CatBoost classifier using positional features.

## 3 Results and discussion

### 3.1 Evaluation metrics

We used five assessment indicators to assess the suggested tool. As discussed in the dataset section, we have used individual chromosomes for testing which do not overlap with the training dataset. Therefore independent testing is used to compute these assessment indicators. Sensitivity (Sen), Specificity (Spe), Accuracy (ACC), F1 Score, and MCC are the figure of merits. These criteria are extensively used in the literature (Rehman *et al.* 2022a, 2021), and we have incorporated them to allow for a fair comparison with current tools. These measures can be stated numerically as, where,


(10) $$\text{Sen}=\frac{\text{TP}}{\text{TP}+\text{FN}},$$

(11) $$\text{Spe}=\frac{\text{TN}}{\text{TN}+\text{FP}},$$

(12) $$\text{ACC}=\frac{\text{TP}+\text{TN}}{\text{TP}+\text{FP}+\text{TN}+\text{FN}},$$

(13) $$F_{1}=\frac{\text{TP}}{\text{TP}+\frac{1}{2}(\text{FP}+\text{FN})},$$

(14) $$\text{MCC}=\frac{\text{TP}\times \text{TN}-\text{FP}\times \text{FN}}{\sqrt{\left(\text{TP}+\text{FN})(\text{TN}+\text{FN})(\text{TP}+\text{FP})(\text{TN}+\text{FP}\right)}},$$

$$\begin{array}{c} \text{TP}\Rightarrow \mathrm{TruePositive}\\ \text{TN}\Rightarrow \mathrm{TrueNegative}\\ \text{FP}\Rightarrow \mathrm{FalsePositive}\\ \text{FN}\Rightarrow \mathrm{FalseNegative} \end{array}$$

These assessment indicators are frequently used to assess the classification performance, subsequently quantifying various aspects of the model. MCC is the most revealing and reliable parameter if both positive and negative classes of the dataset are equally important in the analysis, and if accurately categorizing the present ground truth data samples is equally important in the study (Chicco *et al.* 2021). F1 Score is the harmonic mean of Precision and Recall, and it provides a more accurate assessment of erroneously categorized instances.

### 3.2 Evaluation of different features and classifiers

**Figure 4.**
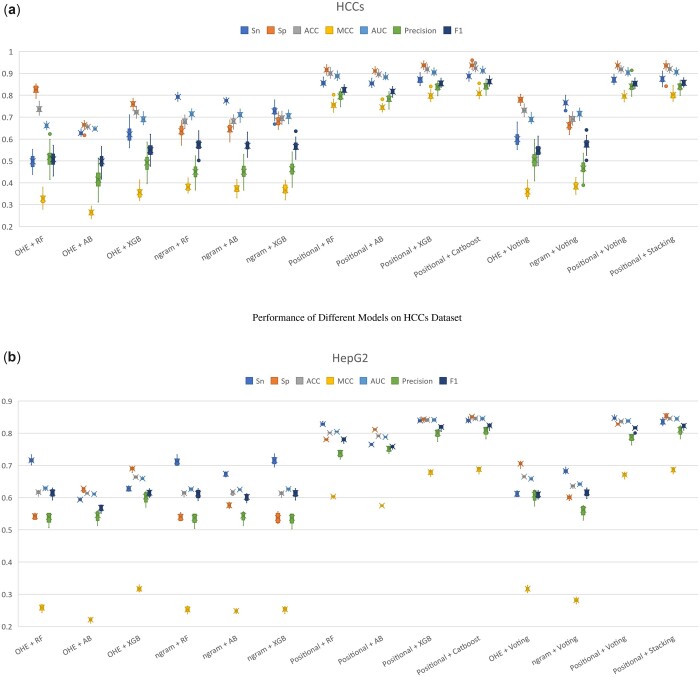
Evaluation of different feature extraction and classification techniques on two adopted benchmark datasets HCCs (**a**) and HepG2 (**b**). The box plot shows the average values and the variation over of all cells in the dataset. Acronyms are; Random Forest (RF), AdaBoost(AB); eXtreme Gradient Boosting (XGB), Sensitivity (Sn), Specificity(Sp), Accuracy (ACC), Matthews correlation coefficient (MCC), and Area Under the Receiver Operating Characteristic Curve (AUC).

**Figure 5.**
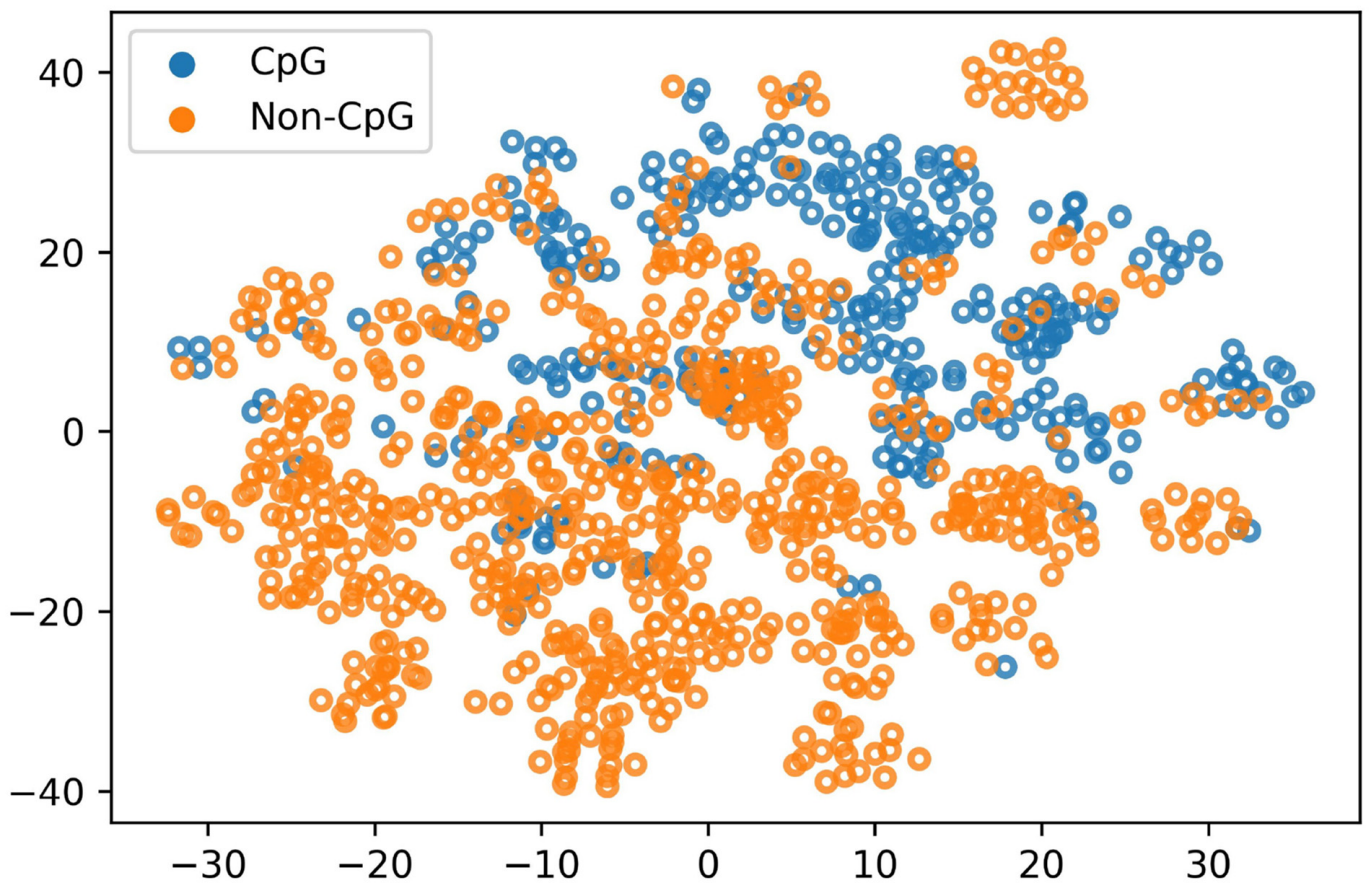
t-distributed stochastic neighbor embedding (t-SNE) positional feature distribution of CpG and Non-CpG sites.

In order to show the supremacy of positional features with respect to encoded features, we have carried out a comparative analysis between them. Moreover, analysis is also carried out between different classifiers. Figure 4 illustrates these analyses. According to both benchmark datasets, characteristics from the positional features have outperformed other feature encoding schemes. Positional features have shown the best performance followed by n-gram and then One-Hot encoding. Figure 5 shows the t-distributed stochastic neighbor embedding plot of positional features where the discrimination between CpG and Non-CpG sites can be observed.

Findings imply that positional features have the potential to greatly improve the performance of CpG site identification. Therefore, we further explored the best classifiers using positional features. Figure 4 illuminates the results achieved by different classifiers being trained using positional features. Among all Catboost classifier is able to achieve better performance. The CatBoost algorithm achieved the maximum average F1-score of 0.8632 for the HCCs dataset and 0.8241 for the HepG2 dataset while the maximum average MCC achieved is 0.8102 and 0.6884 for HCCs and HepG2 datasets respectively. XGB classifier has also showcased good results but during quantitative analysis, it is slightly lower in performance when compared to CatBoost. Figure 4 shows the boxplots of different features with different classifiers, while the cell-wise detailed performance analysis is shown in Supplementary File S1.

In our research, the stacking strategy, which mixes many models to improve predictive performance, did not outperform CatBoost. This result might be related to the unavailability of an adequate and diverse database for training the stacking algorithm. Even though CatBoost is one of the fundamental models in the used stacking strategy, it is vital to remember that stacking adds complexity and interdependence among the models. This intricacy may make it difficult to successfully leverage CatBoost’s strengths inside the stacking architecture.

### 3.3 Evaluation of different ensemble learning techniques

As previously stated, iCpG-Pos is a stacked ensemble model that is created by combining multiple machine learning-based models to construct a more accurate CpG site predictor. To demonstrate the importance of the stacking approach, we compared the effectiveness of the stacking technique used in iCpG-Pos with another prominent ensemble procedure, voting. Results in Supplementary File S1 clearly suggest that the stacking approach has illustrated better results than the voting technique. In some cells, stacking algorithm exhibited improved results than the CatBoost algorithm but in maximum average comparison, CatBoost illustrated better results in the majority of metrics.

### 3.4 Comparison of iCpG-Pos with existing techniques

**Figure 6.**
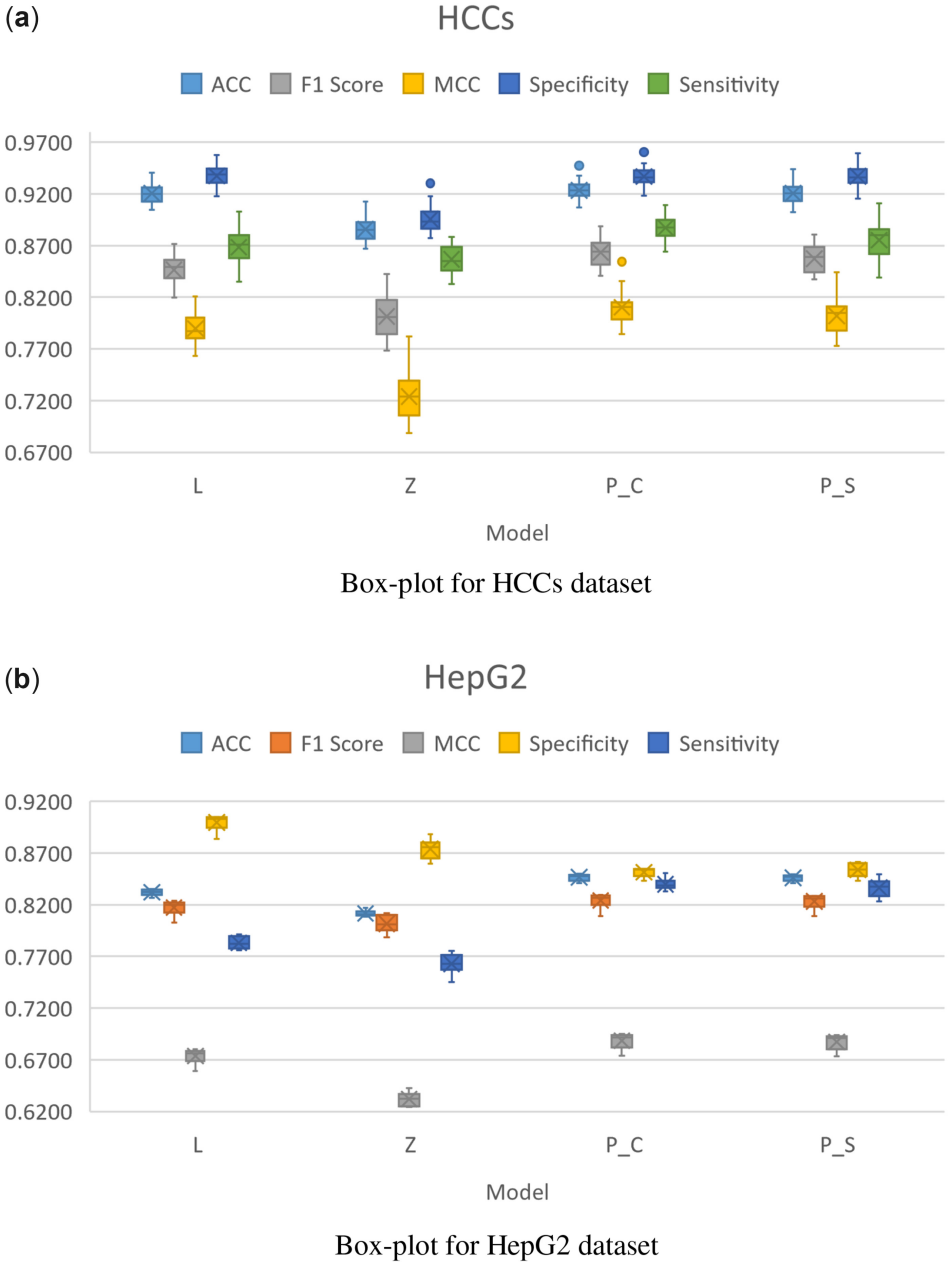
Using the HCCs (a) and HepG2 (b) dataset to illustrate the distribution of evaluation metrics. “L” stands for our LightCpG, “Z” stands for Zhang’s method, “PC” represents proposed algorithm with CatBoost, and “PS” represents proposed algorithm with Stacking framework.

Similar to existing techniques, training and testing dataset chromosomes are used to thoroughly assess and evaluate iCpG-Pos performance to that of existing state-of-the-art approaches. In general, assessing the quality of the model using the same learning and testing dataset is a more objective method for eliminating biasness. As a result, the efficiency of the proposed model is compared to two existing machine learning techniques, including LightCpG and Zhang’s method. Figure 6 represents the box-plot illustrating the distribution of evaluation metrics for different proposed techniques.

As can be seen in Fig. 6a, the proposed CatBoost-based algorithm has shown the best result for all metrics in the case of HCCs dataset. The second best algorithm is the proposed stacking-based model which holds a slight difference from CatBoost-based algorithm. Notably, the F1 score and MCC of the Catboost based algorithm have demonstrated high improvement when compared to both existing models which are LightCpG and Zhang’s algorithm.

Further in Fig. 6b, it can be comprehended that the highest sensitivity, accuracy, MCC, and F1 score is attained by iCpG-Pos architecture using the CatBoost algorithm for the HepG2 algorithm. However, in the case of specificity, the best performance is showcased by the LightCpG algorithm. Moreover, iCpG-Pos architecture with a stacking algorithm has also shown slightly better results than the CatBoost algorithm. But it is important to look into the quantitative difference between the sensitivity and specificity of LightCpG, which is huge. This shows that for the HepG2 dataset, the LightCpG algorithm is comparatively biased towards the Non-CpG class. As represented in Fig. 1 the ratio of unmethylated sites is much larger in the dataset when compared with methylated sites; therefore, the biasness problem is expected in such condition. However, such biasness is not replicated in the case of iCpG-Pos algorithm, interpreting that the proposed algorithm is able to learn the insight features of the CpG sites.

## 4 Conclusion

In this work, we presented the iCpG-Pos technique for enhanced detection of CpG sites. The proposed architecture only uses positional features extracted from the single-cell whole-genome sequencing data. iCpG-Pos presents two techniques which are CatBoost-based and stacking-based. Overall CatBoost-based technique has showcased better results, however, in the case of a few cells stacking-based algorithm illustrated improved results than the CatBoost-based algorithm. All the classification algorithms used in this study are optimized using the OPTUNA framework. An extensive set of experiments have demonstrated that the proposed algorithm serves as a more reliable and efficient predictor than the current technique. This work can be used to uncover the direct linkage between methylation and diseases by comprehending the complicated biological mechanisms that enable methylation.

## Supplementary Material

btad474_Supplementary_Data

## Data Availability

The data underlying this article will be shared on reasonable request to the corresponding author.

## References

[btad474-B1] Akiba T , Sano S, Yanase T et al Optuna: A next-generation hyperparameter optimization framework. In: *Proceedings of the 25th ACM SIGKDD International Conference on Knowledge Discovery & Data Mining, Anchorage AK, USA, August 4–8, 2019*. New York, NY, United States: Association for Computing Machinery, 2019, 2623–31.

[btad474-B2] Angermueller C , Lee HJ, Reik W et al DeepCpG: accurate prediction of single-cell DNA methylation states using deep learning. Genome Biol 2017;18:1–13. 28395661 10.1186/s13059-017-1189-z PMC5387360

[btad474-B3] Bhasin M , Zhang H, Reinherz EL et al Prediction of methylated CpGs in DNA sequences using a support vector machine. FEBS Lett 2005;579:4302–8. 16051225 10.1016/j.febslet.2005.07.002

[btad474-B4] Chicco D , Tötsch N, Jurman G. The Matthews correlation coefficient (MCC) is more reliable than balanced accuracy, bookmaker informedness, and markedness in two-class confusion matrix evaluation. BioData Min 2021;14:13–22. 33541410 10.1186/s13040-021-00244-z PMC7863449

[btad474-B5] Chou K-C. Prediction of protein cellular attributes using pseudo-amino acid composition. Proteins 2001;43:246–55. 11288174 10.1002/prot.1035

[btad474-B6] Clark SJ , Smallwood SA, Lee HJ et al Genome-wide base-resolution mapping of DNA methylation in single cells using single-cell bisulfite sequencing (scBS-seq). Nat Protoc 2017;12:534–47. 28182018 10.1038/nprot.2016.187

[btad474-B7] Das R , Dimitrova N, Xuan Z et al Computational prediction of methylation status in human genomic sequences. Proc Natl Acad Sci USA 2006;103:10713–6. 16818882 10.1073/pnas.0602949103 PMC1502297

[btad474-B8] Di Lena P , Sala C, Prodi A et al Missing value estimation methods for DNA methylation data. Bioinformatics 2019;35:3786–93. 30796811 10.1093/bioinformatics/btz134

[btad474-B9] Ding Y , Tang J, Guo F. Predicting protein-protein interactions via multivariate mutual information of protein sequences. BMC Bioinformatics 2016;17:398. 27677692 10.1186/s12859-016-1253-9 PMC5039908

[btad474-B10] Ernst J , Kellis M. Large-scale imputation of epigenomic datasets for systematic annotation of diverse human tissues. Nat Biotechnol 2015;33:364–76. 25690853 10.1038/nbt.3157 PMC4512306

[btad474-B11] Farlik M , Sheffield NC, Nuzzo A et al Single-cell DNA methylome sequencing and bioinformatic inference of epigenomic cell-state dynamics. Cell Rep 2015;10:1386–97. 25732828 10.1016/j.celrep.2015.02.001 PMC4542311

[btad474-B12] Ganapathiraju M , Weisser D, Rosenfeld R et al Comparative ngram analysis of whole-genome sequences. In: *HLT '02: Proceedings of the second international conference on Human Language Technology Research, San Diego California, March 24–27, 2002*. United States: Morgan Kaufmann Publishers Inc. 2002.

[btad474-B13] Gao D , Zhu B, Sun H et al Mitochondrial DNA methylation and related disease. Adv Exp Med Biol 2017;1038:117–32. 29178073 10.1007/978-981-10-6674-0_9

[btad474-B14] Guo H , Zhu P, Wu X et al Single-cell methylome landscapes of mouse embryonic stem cells and early embryos analyzed using reduced representation bisulfite sequencing. Genome Res 2013;23:2126–35. 24179143 10.1101/gr.161679.113 PMC3847781

[btad474-B15] Horvath S. DNA methylation age of human tissues and cell types. Genome Biol 2013;14:R115–20. 24138928 10.1186/gb-2013-14-10-r115 PMC4015143

[btad474-B16] Hou Y , Guo H, Cao C et al Single-cell triple omics sequencing reveals genetic, epigenetic, and transcriptomic heterogeneity in hepatocellular carcinomas. Cell Res 2016;26:304–19. 26902283 10.1038/cr.2016.23 PMC4783472

[btad474-B17] Jiang L , Wang C, Tang J et al LightCpG: a multi-view CpG sites detection on single-cell whole genome sequence data. BMC Genomics 2019;20:306. 31014252 10.1186/s12864-019-5654-9 PMC6480911

[btad474-B18] Jones PA. Functions of DNA methylation: islands, start sites, gene bodies and beyond. Nat Rev Genet 2012;13:484–92. 22641018 10.1038/nrg3230

[btad474-B19] Kapourani C-A , Sanguinetti G. Melissa: Bayesian clustering and imputation of single-cell methylomes. Genome Biol 2019;20:61–15. 30898142 10.1186/s13059-019-1665-8 PMC6427844

[btad474-B20] Laird PW. Principles and challenges of genome-wide DNA methylation analysis. Nat Rev Genet 2010;11:191–203. 20125086 10.1038/nrg2732

[btad474-B21] Levy JJ , Titus AJ, Petersen CL et al Methylnet: an automated and modular deep learning approach for DNA methylation analysis. BMC Bioinformatics 2020;21:108–15. 32183722 10.1186/s12859-020-3443-8 PMC7076991

[btad474-B22] Liu Z , Xiao X, Qiu W-R et al iDNA-methyl: identifying DNA methylation sites via pseudo trinucleotide composition. Anal Biochem 2015;474:69–77. 25596338 10.1016/j.ab.2014.12.009

[btad474-B23] Pan G , Jiang L, Tang J et al A novel computational method for detecting DNA methylation sites with DNA sequence information and physicochemical properties. Int J Mol Sci 2018;19:511. 29419752 10.3390/ijms19020511 PMC5855733

[btad474-B24] Pavlovic M , Ray P, Pavlovic K et al Direction: a machine learning framework for predicting and characterizing DNA methylation and hydroxymethylation in mammalian genomes. Bioinformatics 2017;33:2986–94. 28505334 10.1093/bioinformatics/btx316 PMC5870843

[btad474-B25] Qiu YL , Zheng H, Gevaert O. A deep learning framework for imputing missing values in genomic data. bioRxiv 2018:406066, preprint: not peer reviewed.

[btad474-B26] Raney BJ , Dreszer TR, Barber GP et al Track data hubs enable visualization of user-defined genome-wide annotations on the UCSC genome browser. Bioinformatics 2014;30:1003–5. 24227676 10.1093/bioinformatics/btt637 PMC3967101

[btad474-B27] Rehman MU , Akhtar S, Zakwan M et al Novel architecture with selected feature vector for effective classification of mitotic and non-mitotic cells in breast cancer histology images. Biomed Signal Process Control 2022a;71:103212.

[btad474-B28] Rehman MU , Tayara H, Chong KT. DCNN-4mC: densely connected neural network based n4-methylcytosine site prediction in multiple species. Comput Struct Biotechnol J 2021;19:6009–19. 34849205 10.1016/j.csbj.2021.10.034 PMC8605313

[btad474-B29] Rehman MU , Tayara H, Chong KT. DL-m6A: identification of n6-methyladenosine sites in mammals using deep learning based on different encoding schemes. IEEE/ACM Trans Comput Biol Bioinform 2022b;20:904–11. 10.1109/TCBB.2022.3192572 35857733

[btad474-B30] Rehman MU , Tayara H, Zou Q et al i6mA-Caps: a capsulenet-based framework for identifying DNA n6-methyladenine sites. Bioinformatics 2022c;38:3885–91. 35771648 10.1093/bioinformatics/btac434

[btad474-B31] Robertson KD. DNA methylation and human disease. Nat Rev Genet 2005;6:597–610. 16136652 10.1038/nrg1655

[btad474-B32] Shensa MJ et al The discrete wavelet transform: wedding the a trous and mallat algorithms. IEEE Trans Signal Process 1992;40:2464–82.

[btad474-B33] Smallwood SA , Lee HJ, Angermueller C et al Single-cell genome-wide bisulfite sequencing for assessing epigenetic heterogeneity. Nat Methods 2014;11:817–20. 25042786 10.1038/nmeth.3035 PMC4117646

[btad474-B34] Stevens M , Cheng JB, Li D et al Estimating absolute methylation levels at single-CpG resolution from methylation enrichment and restriction enzyme sequencing methods. Genome Res 2013;23:1541–53. 23804401 10.1101/gr.152231.112 PMC3759729

[btad474-B35] Stieglitz E , Mazor T, Olshen AB et al Genome-wide DNA methylation is predictive of outcome in juvenile myelomonocytic leukemia. Nat Commun 2017;8:2127–8. 29259179 10.1038/s41467-017-02178-9 PMC5736624

[btad474-B36] Suzuki MM , Bird A. DNA methylation landscapes: provocative insights from epigenomics. Nat Rev Genet 2008;9:465–76. 18463664 10.1038/nrg2341

[btad474-B37] Wan J , Oliver VF, Wang G et al Characterization of tissue-specific differential DNA methylation suggests distinct modes of positive and negative gene expression regulation. BMC Genomics 2015;16:49–11. 25652663 10.1186/s12864-015-1271-4 PMC4331481

[btad474-B38] Whitaker JW , Chen Z, Wang W. Predicting the human epigenome from DNA motifs. Nat Methods 2015;12:265–72, 7 p following 272. 25240437 10.1038/nmeth.3065 PMC4344378

[btad474-B39] Yan Q , Zhou X, Xue W et al Advances in the relationship between epigenetic DNA methylation and histone modification with diseases. Med Recapitulate (China) 2017;23:3160–3.

[btad474-B40] Yu F , Xu C, Deng H-W et al A novel computational strategy for DNA methylation imputation using mixture regression model (MRM). BMC Bioinformatics 2020;21:552–17. 33261550 10.1186/s12859-020-03865-z PMC7708217

[btad474-B41] Zhang W , Spector TD, Deloukas P et al Predicting genome-wide DNA methylation using methylation marks, genomic position, and DNA regulatory elements. Genome Biol 2015;16:14–20. 25616342 10.1186/s13059-015-0581-9 PMC4389802

[btad474-B42] Zhou X , Li Z, Dai Z et al Prediction of methylation CpGs and their methylation degrees in human DNA sequences. Comput Biol Med 2012;42:408–13. 22209047 10.1016/j.compbiomed.2011.12.008

[btad474-B43] Zou LS , Erdos MR, Taylor DL et al; McDonnell Genome Institute. Boostme accurately predicts DNA methylation values in whole-genome bisulfite sequencing of multiple human tissues. BMC Genomics 2018;19:390–15. 29792182 10.1186/s12864-018-4766-y PMC5966887

